# Archaea in and on the Human Body: Health Implications and Future Directions

**DOI:** 10.1371/journal.ppat.1004833

**Published:** 2015-06-11

**Authors:** Mor N. Lurie-Weinberger, Uri Gophna

**Affiliations:** Department of Molecular Microbiology and Biotechnology, George S. Wise Faculty of Life Sciences, Tel Aviv University, Tel Aviv, Israel; Duke University Medical Center, UNITED STATES

Although they are abundant and even dominant members of animal microbiomes (microbiotas), from sponges and termites to mice and cattle, archaea in our own microbiomes have received much less attention than their bacterial counterparts. The fact that human-associated archaea have been relatively little-studied may be at least partially attributed to the lack of any established archaeal human pathogens [1,2]. Clinically oriented microbiology courses often do not mention archaea at all, and most medical school and biology students are only aware of archaea as exotic extremophiles that have strange and eukaryotic-like molecular machinery. Since archaea have been known to be associated with the human gut for several decades, one would think that human microbiome studies may unravel new facets of archaea–human interactions. However, adequate universal primers that amplify both bacterial and archaeal small 16S rRNA genes but not any host rRNA genes were only published in mid-2011 [3], and thus, many studies chose to focus on bacteria alone rather than multiply effort and expense to cover taxa that are considered secondary in importance, if not altogether rare. Here, we provide a brief overview of what is currently known about archaea in and on the human body and their potential effects on human health (for additional reviews on archaea and their potential involvement in human disease, see [4–8]).

## Archaea in the Human Gut

**Fig 1:**
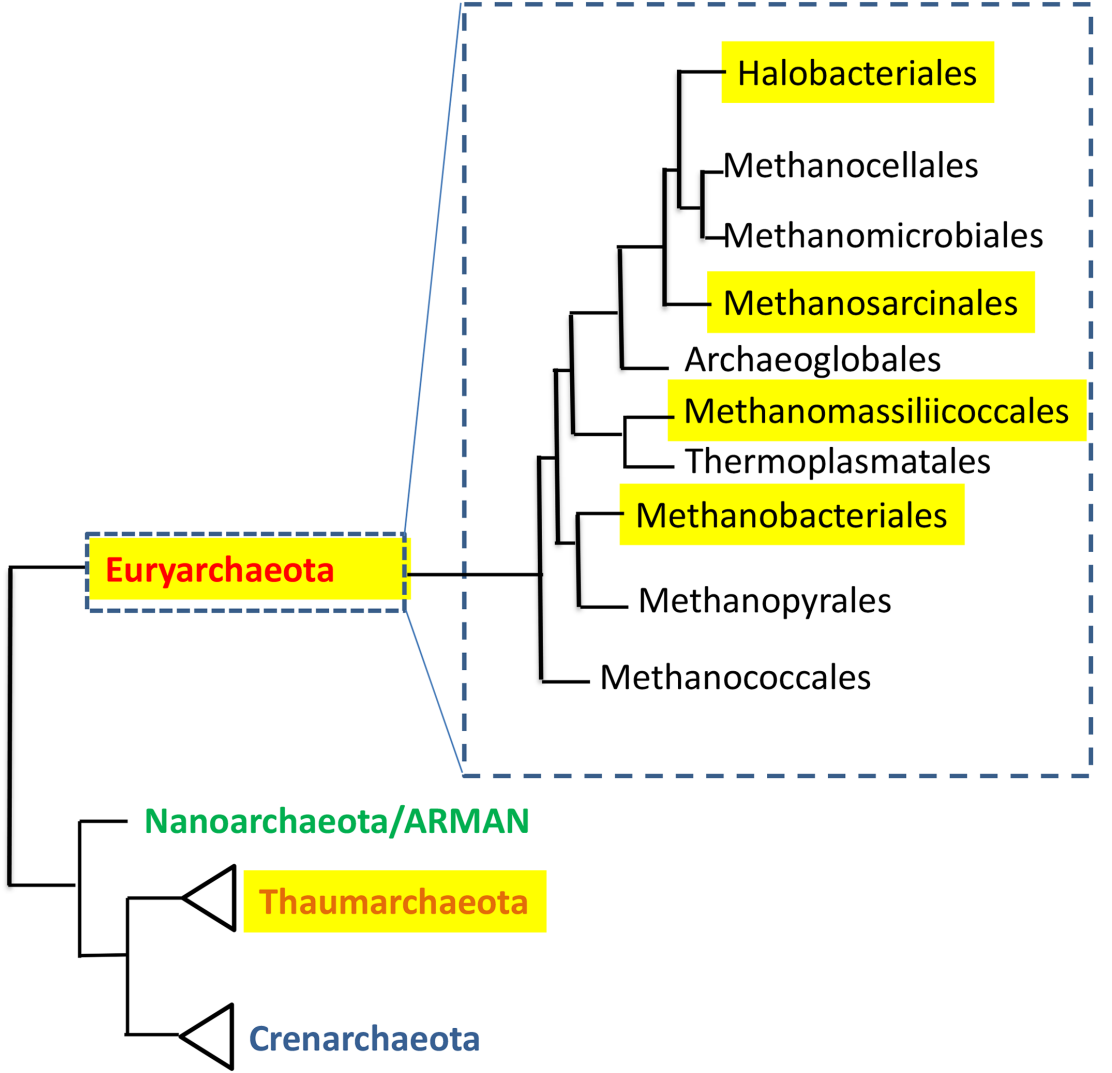
The distribution of human-associated archaea in the phylogenetic tree of the domain Archaea. Tree is based on [63], and [64]. Highlighted are groups that contain human-associated members.

The human large intestine (colon), in healthy individuals, has extremely low oxygen concentrations, and over 90% of its microbiota are strict anaerobes. Researchers taking metagenomic fecal microbiota surveys of adult Europeans could assign about 0.8% of the genes in their dataset to archaea [9], and similar numbers (0.2%–0.3%) were reported for Amerindians and Malwaians [10], while North Americans had much lower fractions (<0.05%). With the exception of a single report indicating the presence of halophilic archaea in biopsies of inflammatory bowel disease patients [11], archaea that reside in the human colon are nearly always methanogens. Most of these strict anaerobes belong to the order Methanobacteriales (Fig 1), the most common genera being the closely related *Methaonbrevibacter* and *Methanosphaera*. *Methanobrevibacter* (previously called *Methanobacterium*) was first isolated from human stool as early as 1968 [12], followed nearly 15 years later by the discovery that such fecal isolates belonged to the species *Methanobrevibacter smithii*. *M*. *smithii* has been shown to be present in up to 95.7% of human subjects [13], and to be the most abundant methanogen in the human gut by several studies, comprising up to as much as 10% of all anaerobes found in a healthy individual's colon [14–16]. Remarkably, its abundance appears to remain stable over time, even following radical dietary changes [17], and it is highly heritable, meaning that monozygotic twins are more concordant for its presence, or absence, than dizygotic twins [18,19]. Importantly, substrates for methanogenesis, such as H_2_, methanol and acetate, are mostly derived from the end products of bacterial fermentation. The second most abundant methanogen in this environment is *Methanosphaera stadtmanae*. This organism, which has the most restricted energy metabolism of all known methanogenic archaea, is totally dependent on acetate as a carbon source, and its methane production requires methanol and hydrogen [20]. The human colon and other mammalian intestines are dominated by hundreds of bacterial species [14], and it is therefore not surprising to observe that the genomes of *M*. *smithii* and *M*. *stadtmanae* appear to be very rich in inter-domain lateral gene transfers, especially relating to glycosyltransferases and ABC transporters in both species and adhesin-like proteins in *M*. *smithii* [21,22]. These laterally acquired genes are thought to have played a significant role in these organisms' initial adaptation to mammalian hosts [21,22]. Both of these species have been recently shown to induce monocyte-derived dendritic cell maturation, and *M*. *stadtmanae* also induced a strong pro-inflammatory cytokine release from these cells [23] and is more prevalent in patients with inflammatory bowel disease [24].

In ruminants, presence of the methanogen *Methanobrevibacter ruminantium* can result in loss of up to 6% of all ingested energy [25]. In contrast, it has been suggested, based primarily on mouse studies [26,27], that gut methanogens contribute to human obesity. Indeed, methanogens are capable of syntrophic interactions with bacteria that enhance production of short-chain fatty acids, which provide a considerable caloric contribution to the host. However, more recent evidence from several large human studies strongly supports an association of *M*. *smithii* with leanness [19,28–30]. Future research may determine more precisely the roles that methanogens play in host metabolism in order to enable new microbiota-based approaches for weight management.

Another possible connection between gut methanogens and human health is the strong association between methanogen presence and chronic constipation [31]. Methane was shown to slow intestinal transit time by 59% [32], and thus may contribute substantially to constipation. However, a shorter intestinal transit time probably selects against the presence of methanogens, since they tend to have generation times that are longer than those of many gut bacteria, even when grown in the most favorable, state-of-the-art culture media [33]. In agreement with these in vitro data, human studies have shown a lower prevalence of methanogens (determined by methane excretion) in patients that tend to have diarrhea episodes (such as those with inflammatory bowel diseases) compared to healthy individuals [34]. Taken together, these findings indicate that in individuals with already slow intestinal transit, methanogens may bloom and promote further constipation.

Until recently, there were only six known orders of methanogens, only one of which (the above-mentioned Methanobacteriales) was represented in the human body. However, recent reports show that there is a seventh order, Methanomassiliicoccales, which includes the gut-residing methanogens *Methanomassiliicoccus luminyensis* and Candidatus Methanomethylophilus alvus [35]. Both these organisms are methylotrophic archaea isolated from human feces [36,37]. Although *M*. *luminyensis* has been shown to require H_2_ for methanogesis, it can utilize not just methanol but also trimethylamine for growth in the presence of H_2_, which has important implications for human health [38]. Trimethylamine is a metabolite produced from host dietary choline [39] or carnitine [40], by the gut microbiota, which is later oxidized to trimethylamine N-oxide (TMAO) by the host enzyme flavin monooxygenases. TMAO has been shown to promote atherosclerosis in mice and to be a strong biomarker for human cardiovascular disease [39]. Thus, having an archaeal community that may remediate not just methanol toxicity, but also prevent trimethylamine accumulation and TMAO production could be highly desirable. Accordingly it has been suggested that these archaea can be used as "archaebiotics," for the prevention of cardiovascular disease as well as trimethylaminuria, a hereditary deficiency in flavin monooxygenases activity that results in an unpleasant fishy odor in breath and sweat [38]. Whether this novel and exciting therapeutic concept can be tested in animals and subsequently translated into the clinic remains to be seen.

## Subgingival Archaea

Methanogenic Archaea have been reported in subgingival dental plaque as early as 1987 [41]. To date, three genera have been successfully isolated from subgingival plaque: *Methanobrevibacter* [42,43], *Methanosphaera* (based on weak antigenic similarity) [44], and *Methanosarcina* (based on physiology and staining) [45]. Additionally, 16S rRNA gene amplicon sequencing studies detected archaea related to Thermoplasmata [46,47] (which, in retrospect, probably belong to the seventh order of methanogens; see gut methanogens section, above), as well as members of the *Methanobacterium* genus [48,49]. In general, it appears that the genetic diversity of archaea of the human subgingival dental plaques is low, much as is the case for the gut methanogens, and that *Methanobrevibacter oralis* is by far the most prevalent methanogen found in this environment. In a recent review, Nguyen-Hieu et al. pooled the data from several studies of methanogens in the oral cavity and concluded that *M*. *oralis* is significantly associated with periodontal disease both in terms of abundance comparisons between patients and controls and between diseases and healthy sites within the same patient [50]. Furthermore, they concluded that indirect evidence supports the contribution of that methanogen to periodontal disease and that this contribution likely stems from syntrophic interactions with sulfate-reducing bacteria. Thus, a mixed infection may be required for a direct causal demonstration of the pathogenic contribution of *M*. *oralis* in an animal model of periodontal disease. Unlike many antibiotics that do not target archaea (because they do not have a peptidoglycan cell wall and have ribosomes that are more eukaryotic like [51]), metronidazole, which is commonly used to treat periodontitis, is highly effective against *M*. *oralis* [52] and, thus, suppression of *M*. *oralis* could contribute to its efficacy [50]. Statins that inhibit the activity of 3-hydroxy-3-methylglutaryl coenzyme A reductases of eukaryotes and archaea lower blood cholesterol in humans, but they also effectively inhibit archaeal growth because they block the synthesis of their main membrane lipids [53]. If indeed *M*. *oralis* is a "co-pathogen," it would be interesting to examine the effects of "archaea-specific" drugs such as statins on periodontal disease, for example, by examining the periodontal pockets of patients who have recently been prescribed statins before and after several months of statin use.

## Archaea on the Human Skin

Archaea on the human skin have been discovered only in recent years. A 16S rRNA gene amplicon sequencing study focusing on the navel found rare occurrence of *Methanobrevibacter* in several individuals. Even more rare were phylotypes belonging to the halophilic archaea (family Halobacteriaceae), which were only present in a single individual, who abstained from showers or baths for several years prior to sampling [54]. A large metagenomic survey detected reads that matched archaea in most individual samples, but all archaeal sequences combined did not exceed 2.3 × 10^–5^ of the reads in any sample [55]. A recent study, using archaeal-specific 16S rRNA gene primers, found archaea to be present on the skin of 13/13 volunteers, with relative abundances that exceeded 4% in one individual. Five out of five individuals that were more closely studied displayed human-associated archaea that were not methanogens, as may be expected in such an aerobic niche. Instead, the dominant skin-associated archaea belonged to the phylum Thaumarchaeota [56]. In a study that continuously sampled the skin (left and right palm) of one male and one female over several months, the male had only transient Thaumarchaeota, while the female had persistent, albeit low, presence of these archaea on her right palm [57], indicating there is likely to be high inter-individual variation in skin colonization by these archaea. Like other members of the Thaumarchaeota phylum, skin phylotypes are thought to be chemolithotrophic ammonium oxidizers and encode characteristic *amoA* gene homologs [56]. Whether the relatively small amounts of ammonium in sweat are sufficient to sustain such metabolism in the human skin is unclear, but ammonium release in sweat was shown to increase during physical exercise [58] and could reach several mM [59]. Thus, people who sweat and/or exercise more could harbor larger communities of these archaea.

## Concluding Remarks

The availability of reference genomes from previously unrepresented groups, such as the Methanomassiliicoccales for metagenomic analysis, as well as better 16S rRNA gene primers, should improve the detection of archaea in human microbiome studies. This improvement is highly timely, since archaea are still an under-detected and little-studied part of the human microbiome, and their contributions to human health or disease remain mostly unknown. This knowledge gap should be addressed in the near future to inform clinicians, many of whom are totally unaware of these organisms. While no human clinical study studying the in vivo effects of statins on archaea in our microbiomes has been published, in vitro results [60] strongly suggest that these drugs could inhibit the growth of archaea in the human body. While the inhibitory concentrations reported for archaea in vitro (4 mg/L, about 10 μmol/L for lovastatin [60]) are much higher than their level in circulation (9.4 nmol/L [61]), their levels in the gut may be very much higher. Moreover, in highly competitive niches, such as the colon, even partial growth inhibition may cause extinction. In developed countries, such as the United States, statin use is on the rise, and over a third of people over 65 use these drugs for their cholesterol-lowering effects, unaware that at the same time they are taking a broad-spectrum anti-archaeal agent. At the moment, there is little evidence of whether eradication of human-associated archaea (and potentially their bacterial syntrophs) will be beneficial or harmful for human health, with the possible exception of periodontal disease. Thus, before archaea become part of the "disappearing human microbiota" [62] we should at least know if we are going to miss them when they are gone.
